# The Short-Term Load Forecasting Using an Artificial Neural Network Approach with Periodic and Nonperiodic Factors: A Case Study of Tai'an, Shandong Province, China

**DOI:** 10.1155/2021/1502932

**Published:** 2021-10-26

**Authors:** Jiuyun Sun, Huanhe Dong, Ya Gao, Yong Fang, Yuan Kong

**Affiliations:** College of Mathematics and Systems Science, Shandong University of Science and Technology, Qingdao 266590, China

## Abstract

Accurate electricity load forecasting is an important prerequisite for stable electricity system operation. In this paper, it is found that daily and weekly variations are prominent by the power spectrum analysis of the historical loads collected hourly in Tai'an, Shandong Province, China. In addition, the influence of the extraneous variables is also very obvious. For example, the load dropped significantly for a long period of time during the Chinese Lunar Spring Festival. Therefore, an artificial neural network model is constructed with six periodic and three nonperiodic factors. The load from January 2016 to August 2018 was divided into two parts in the ratio of 9 : 1 as the training set and the test set, respectively. The experimental results indicate that the daily prediction model with selected factors can achieve higher forecasting accuracy.

## 1. Introduction

Electricity load forecasting has always been a very critical part of electricity system operation. Based on electricity load forecasting, many operating decisions can be made, such as generating capacity, reliability analysis, and maintenance plan [1]. Therefore, it has been an important task to improve the accuracy of load forecasting. If the load forecasting results is inaccurate, it may lead to the fact that social electricity demand cannot be met or energy waste [2]. However, if the demand for electricity loads can be accurately predicted, it can improve energy utilization rate and safeguard the operation of the power system.

In recent years, many electricity load forecasting models have been proposed. Generally speaking, these models can be classified into three major categories: time series models, artificial intelligence models, and hybrid models [3]. At the early stage of electricity load forecasting, time series models were mainly used [4], such as linear regression [5], the autoregressive integrated moving average models [6–10], exponential smoothing models [11, 12], and Kalman filtering [13]. These models are effective in linear prediction problems but cannot handle complex nonlinear load time series perfectly [4]. It is known that the load series are nonlinear functions of the exogenous variables [14]. Therefore, artificial intelligence models that can handle the nonlinear functions are used for electricity load forecasting. Artificial intelligence models commonly used include artificial neural network (ANN), support vector machine (SVM), bagged regression trees [15], and random forest [16]. The application of ANN for load forecasting started in the late 1980s and early 1990s [17, 18]. Since then, a large number of ANN-based prediction models have been proposed, such as extreme learning machine (ELM) [19] and LSTM [20, 21]. This shows that the ANN's property of fitting any function can contribute to electricity load forecasting. SVM is another model suitable for electricity load forecasting [22]. To overcome the drawbacks of large amount of data and slow processing speed when constructing SVM, researchers have combined SVM with other algorithms, such as the ant colony algorithm [23] and the simulated annealing algorithm [24]. In addition, many hybrid models integrating different models are proposed [1, 25, 26]. The hybrid model may be superior to the single model because the shortcomings of the single model are overcome to some extent [3]. Among these models, ANN has received the largest share of attention [14]; and the forecasting systems based on ANN have been well accepted in practice [27].

Although many excellent models have been proposed, most researchers do not focus on optimizing the input-output feature or doing a feature selecting work [4]. Since the load series are nonlinear functions of the exogenous variables, selecting features from influencing factors such as society and weather is an essential step in electricity load forecasting [28, 29]. Better results can be obtained if these models can establish the relationship between load and the exogenous variables. However, the influence of the influencing factors is not easily expressed in mathematical formulas, and the influencing degree is not easily quantified. In order to make full use of each influencing factor, ANN is used as the prediction model in this paper.

The remaining sections of this paper are as follows.  Section 2 focuses on the basic structure of ANN.  Section 3 analyzes the electricity load data, and six periodic factors and three nonperiodic factors are selected.  Section 4 designs five sets of comparison experiments based on the predictors and analyzes the results.  Section 5 summarizes the work of this paper and introduces the next research directions.

## 2. Artificial Neural Network

### 2.1. The Basic Structure of ANN

**Figure 1:**
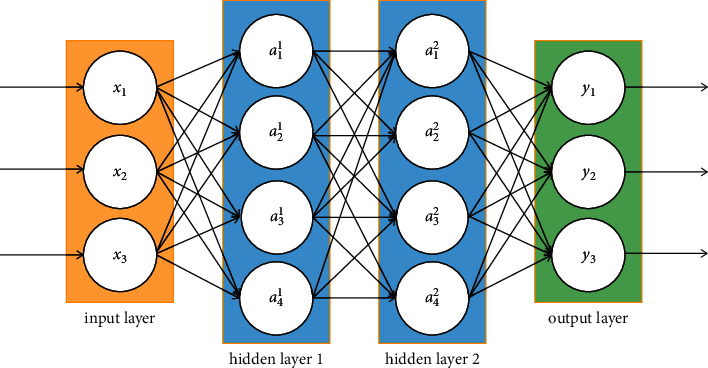
The structure of a four-layered BPNN.

Neural network has a strong self-learning ability whereby the transformation from the input to the output is learned and not programmed [30]. A widely used model called backpropagation neural network (BPNN) is shown in Figure 1. The neural network is divided into three layers: one input layer, one or multiple hidden layers, and one output layer. Each layer consists of a number of neurons and each neuron in one layer is connected to neurons in adjacent layers with different weights. The signals flow into the neural network through the input layer, pass through each hidden layer in turn, and finally arrive at the output layer [18]. Then, the network iteratively adjusts the connection weights through backpropagation to reduce the error between the output and the actual value.

### 2.2. Forward Propagation Algorithm of ANN

Forward propagation is the first step of BPNN. Each neuron takes the output of the previous layer, stacks it with different weights, adds the bias, and then processes it through the activation function to get its own output. We have

(1) $$\begin{array}{c} a_{k}^{l}=f\left(\sum_{i=1}^{K_{l-1}}a_{i}^{l-1}\omega_{ik}^{l}+b_{k}^{l}\right), \end{array}$$

(2) $$\begin{array}{c} f\left(x\right)=\frac{1}{1+e^{-\alpha x}}, \end{array}$$

where *a*_*k*_^*l*^ is the output of the *k-*th neuron in the *l-*th layer and *f* is the activation function. The role of the activation function is to add a nonlinear influence to the neural network. There are many types of activation functions, such as Sigmoid, tanh, and ReLU. The Sigmoid is used as the activation function of BPNN in this study, as in equation (2). *ω*_*ik*_^*l*^ is the connection weight between the *a*_*i*_^*l*−1^ neuron and the *a*_*k*_^*l*^ neuron, and *b*_*k*_^*l*^ is the deviation of the *k-*th neuron in the *l-*th layer.

### 2.3. Backpropagation Algorithm of ANN

The backpropagation is actually an optimization process whose goal is to minimize the error between the output and the target. The training process is to iteratively adjust the values of weight *ω* and bias *b*. Assuming that the target is *y* and the output of the neural network is $\hat{y}$, the function that measures the error of the target and the neural network is the loss function (*L*), as in equation (3). The loss function includes mean squared difference loss function and cross entropy loss function.

(3) $$\begin{array}{c} L=\sigma \left(y,\hat{y}\right). \end{array}$$

Since the output *y* is calculated from each hidden layer, *L* is a function of *ω*, *b*. The gradient of *L* over *ω* gives Δ*ω* and Δ*b*, which gives new *ω* and *b*.

## 3. Data Processing and Analysis

In this section, the raw data is preprocessed. Then, the features which help to improve the accuracy of electricity load forecasting are extracted by analyzing the preprocessed data.

### 3.1. Data Processing

The original data comes from the hourly electricity load data of Tai'an City, Shandong Province, China, from January 1 of 2016 to August 31 of 2018. The main work of preprocessing is to fill in missing values and remove outliers.

**Figure 2:**
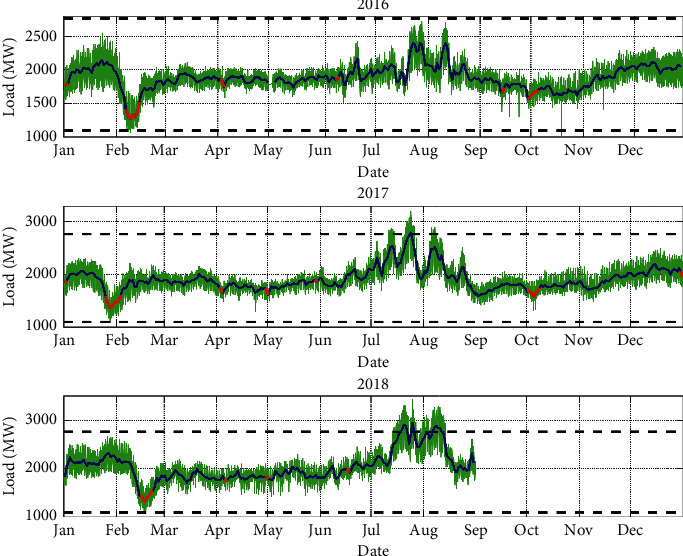
Original load profile from January 1, 2016, to August 31, 2018, in Tai'an city. The green fluctuation line represents the load observed at one-hour interval, the blue curve represents the daily average load, the red curve represents the load on national legal holidays, and the dotted line represents the boundary of Pauta criteria: *μ*+3*σ* and *μ* − 3*σ*.

There are 106 groups of missing values in the original data, accounting for 0.45% of the total. Among them, 57 missing values are from 1 : 00 : 00 on May 1 of 2016 to 9 : 00 : 00 on May 3 of 2016. Since this period is May Day, its change trend is not consistent with the usual. These 57 missing values are not filled (Figure 2). At the same time, the other 49 missing values randomly distributed in the original data were filled in by linear interpolation.

Outliers are identified by Pauta criteria. For data *x*_*i*_,

(4) $$\begin{array}{c} \left|x_{i}-\mu \right|>3\sigma , \end{array}$$

where *x*_*i*_ is an outlier. *μ* is the mean and *σ*is the variance of the original data. Outliers appear in four places.

1. The summer of 2017 and 2018. Due to the large changes in the electricity load every summer, these data remain unchanged.
2. The Chinese Lunar Spring Festival in 2016. Since there is a drop in electricity load during the Spring Festival every year, these data remain unchanged.
3. At 7 : 00 : 00 on October 21, 2016. The original data are deleted and filled with linear interpolation.

### 3.2. Data Analysis

The changing pattern and influence factors can be obtained by analyzing the raw load time series, which will effectively improve the accuracy of short-term load forecasting. As can be seen in Figure 2, the pattern of load variation is essentially the same from year to year. The loads in winter and summer are higher than those in spring and autumn, while load variation is more dramatic in summer. By observation of the daily averaged load (the blue line), it shows the weekly pattern; similarly, the hourly load variation (the green line) demonstrates the daily variation. In addition, the load level decreases during holidays (the red line). Especially during the Chinese Lunar Spring Festival, the drop is very significant.

#### 3.2.1. Spectrum Analysis

**Figure 3:**
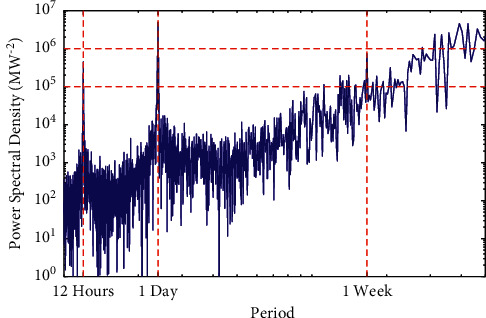
Power spectral density of load.

To verify the daily and weekly variations in Figure 2, the electricity loads are analyzed using spectral analysis. In Figure 3, there are three main peaks. The highest peak occurs when the period is one day. This indicates that the daily variation of the load is most pronounced. The second peak occurs when the period is one week. This indicates that weekly variation is also evident. This is consistent with the observation in Figure 2. In addition, a peak occurs at 12 hours, which may be due to the diurnal variation of the load.

#### 3.2.2. Average Hourly Load Analysis of Each Day

The spectrum analysis shows that the daily variation of the electricity load is an important feature. It is necessary to analyze the daily variation of the hourly average load.

**Figure 4:**
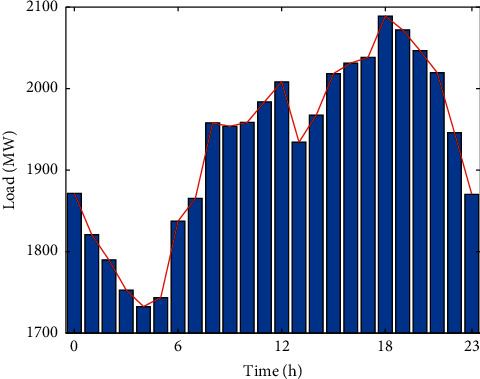
Average hourly load of each day.

From Figure 4, the daily variation in hourly average load shows two rises and two falls. 5 : 00–12 : 00 is the first rising period, and the first fall occurs after 12 : 00 due to lunch. 13 : 00–19 : 00 shows the second rise in load, but the magnitude of this rise is smaller than that of the first rise. After 18 : 00, the load decreases for the second time due to the end of work, and it decreases until 5 : 00 the next day. In general, the daytime load basically kept rising, while the night load kept falling.

#### 3.2.3. Average Hourly Load Analysis of Each Week

According to Figures 2 and 3, the weekly variation of the electricity load is another important feature. From Figure 6, the variation of electricity load for each day of the week is basically the same. There is a large difference in electricity load from 6 : 00 to 18 : 00. From Figure 6, the electricity load on Wednesday, Thursday, and Friday stays at a higher level, and the electricity load on Sunday is the smallest value in the week. This makes the variation from Saturday to the following Monday larger. The reason is that the weekend is a nonworking day.

#### 3.2.4. Average Hourly Load Analysis of Each Season

It can be seen from Figure 2 that the variation of electricity load is not the same in different seasons. Since Tai'an is located in northern China, its seasonal division is consistent with the seasonal division method of the Chinese lunar calendar. In the Chinese 24 solar terms, the Beginning of Spring, the Beginning of Summer, the Beginning of Autumn, and the Beginning of Winter are used for dividing the seasons. The dates of the four solar terms are around February 4, May 5, August 7, and November 7 of each year.

Figure 7 shows that the summer and winter loads are higher compared to the spring and fall loads. Most of the loads in the spring and autumn are below the average. From Figure 8, the average hourly load varies greatly in different seasons. In addition, the variations of electricity load in summer and autumn were similar, and the variations in winter and spring were similar.

### 3.3. Features Selection

To improve the accuracy of electricity load forecasting, it is also necessary to consider the characteristic factors that have an impact on the power load. In the following, the main characteristic factors are selected through the analysis of electricity data load.

The power spectrum analysis and the analysis of the daily variation of the hourly average load prove that the daily variation is a significant feature. The hour of the day and the loads of 24 hours before the forecast moment are considered as a set of predictors.

Similarly, since the weekly variation is another important feature, the day of the week, the load at the same moment from previous week, and the total load on the same day from previous week are considered as a set of predictors.

By analyzing the electricity load in different seasons, the season is one of the factors. There are many manifestations of seasons, such as temperature, humidity, wind power, and wind direction. The correlation analysis of electricity load with these factors shows that the correlation between temperature and load is 0.61 (it is generally considered that the correlation is 0.3–0.8), and the rest are less than 0.2. Therefore, the season and the temperature of the previous moment are considered as a set of predictors.

In addition, the electricity load decreases during weekends and holidays, which are nonworking day moments. So, weekends and holidays are considered as a set of predictors.  Table 1 shows the holidays for the years 2016–2018.

## 4. Experiments and Results Analysis

### 4.1. Dataset and Evaluation Criterion

The load data from January 2016 to August 2018 in Tai'an are divided into two parts, the training set and the test set. The ratio of the training set to the test set is 9 : 1. It means 10% of the overall data is randomly selected as the test set and the remaining part is used as the training set.

Three evaluation criterion indexes are used to explore the accuracy of predicted results: the mean absolute (MAE) mentioned in equation (5), the mean percentage error (MPE) mentioned in equation (6), and the MAPE mentioned in equation (7). MAE can describe the amount of deviation between actual values and predicted values, while MPE and MAPE can reflect the degree of deviation [31]. The three equations are as follows:

(5) $$\begin{array}{c} \text{MAE}=\frac{1}{N}\overset{N}{\underset{i=1}{\sum }}\left|y_{i}-{\hat{y}}_{i}\right|, \end{array}$$

(6) $$\begin{array}{c} \text{MPE}=\frac{1}{N}\overset{N}{\underset{i=1}{\sum }}\frac{y_{i}-{\hat{y}}_{i}}{y_{i}}, \end{array}$$

(7) $$\begin{array}{c} \text{MAPE}=\frac{1}{N}\sum_{i=1}^{N}\left|\frac{y_{i}-{\overset{⌢}{y}}_{i}}{y_{i}}\right|\times 100\%, \end{array}$$

where *N* refers to the total number of test set; *y*_*i*_ refers to the actual load value at time point *i*; and ${\hat{y}}_{i}$ refers to the predicted load value at time point *i*.

### 4.2. Experimental Design

In order to verify the role of the selected predictors in short-term electricity load forecasting, one set of basic experiments *S* and four sets of comparison experiments *S*1, *S*2, *S*3, and *S*4 are designed in this paper. The specific design is as follows:

(1) *Basic Experiments S*. The hour of the day and the loads of 24 hours before the forecast moment are used as inputs
(2) *Comparison Experiments S*1. Based on *S*, the day of the week, the load at the same moment from previous week, and the total load on the same day from previous week are added to the input
(3) *Comparison Experiments S*2. Based on *S*, the season and the temperature of the previous hour are added to the input
(4) *Comparison Experiments S*3. Based on *S*1, the weekday factor and the holiday factor are added to the input
(5) *Comparison Experiments S*4. Based on *S*3, the season and the temperature of the previous hour are added to the input

### 4.3. Results Analysis and Discussion

The MAE, MPE, and MAPE values for the five sets of experiments are shown in Table 2. The basic experiment *S* has the largest MAE and MAPE. The reduction of MAE and MAPE shows that the prediction accuracy was improved to different degrees after adding the predictors. This indicates that the selected predictors are beneficial for short-term electricity load forecasting. Meanwhile, the performance of the three evaluation criterion indexes that the basic experiment *S* is reasonable.

It is worth noting that although the values of MPE for each experiment are small, the variation is not significant. The reason is that the deviations between actual and predicted values are not absolutized, leading to a situation where the positives and negatives cancel each other out. Therefore, the effects of different predictors on electric load forecasting will be discussed based on the MAPE that can better reflect the actual situation of forecast value errors.

Figure 9 shows the comparison of the predicted and actual values of the hourly average load for each day of the test set. In general, the predicted values of the five groups of experiments are close to the actual values. The electricity load is significantly different from the actual value only at 12 : 00 : 00, 13 : 00 : 00, and 18 : 00 : 00. Because 12–13 is lunch time, the originally rising of electricity load has a downward trend. Similarly, 18 : 00 : 00 is generally the end of the work time, which also makes the electricity load drop. The rapid variation in load in the three moments leads to a slightly larger error.

**Figure 5:**
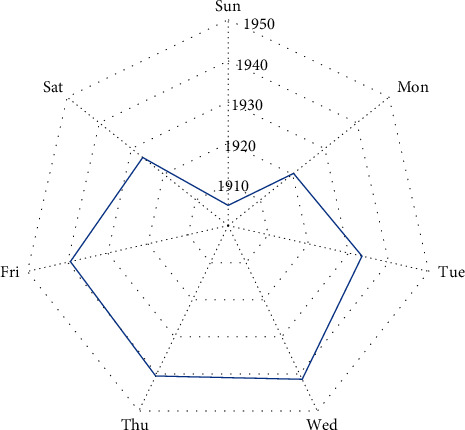
Average hourly load of each week.

**Figure 6:**
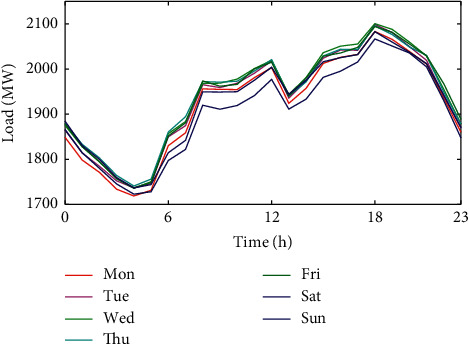
Average daily total load of each week.

**Figure 7:**
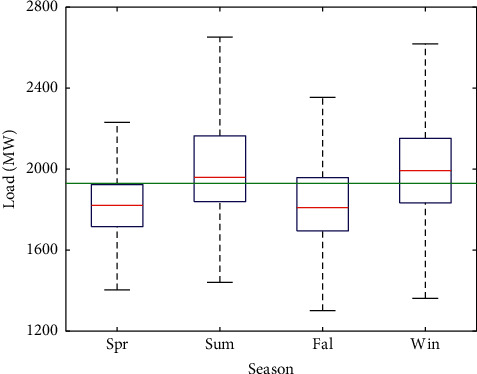
Boxplot of seasonal load distribution. The green line represents the average value of the overall load.

**Figure 8:**
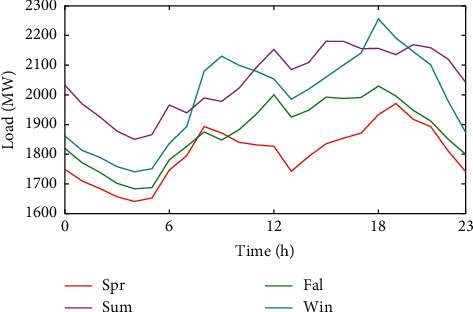
Average hourly load of each season.

**Figure 9:**
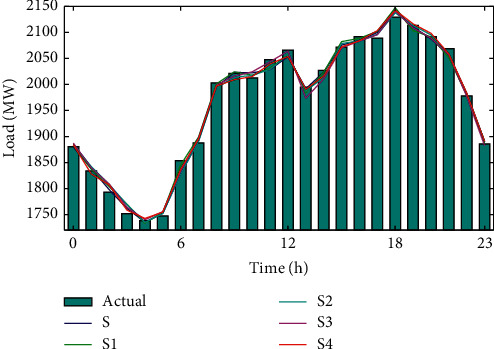
Comparison for average hourly load of each day between actual and forecast loads.

**Figure 10:**
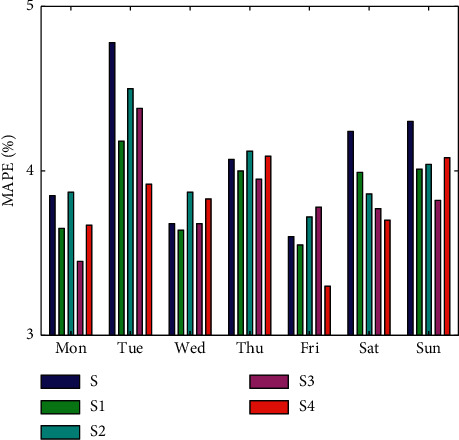
Average MAPE of all five models for the seven days of the week.

Figure 10 shows the averaged MAPE for the five experiments from Monday to Sunday. It can be seen that the MAPE is lower on Wednesday, Thursday, and Friday when the load is more stable. In Figure 5, it can be seen that the load goes through a process of falling and then rising from Saturday to Tuesday. The huge variation in load is the main reason for the higher MAPE in these days. After considering nonworking days and working days separately, there is a significant decrease in MAPE for Monday, Saturday, and Sunday. In other words, considering whether it is a weekday has an effect only on weekends and Monday. This indicates that the prediction accuracy of nonworking days is more easily improved from the weekday factor.

**Figure 11:**
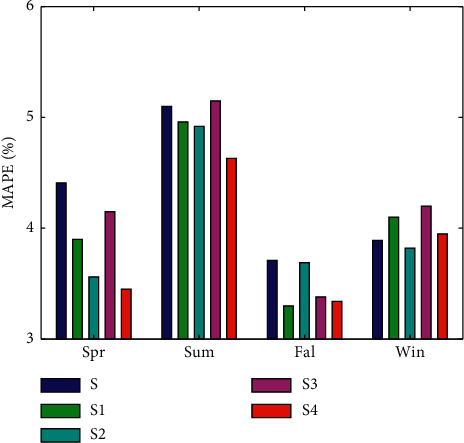
Average MAPE of all five models for each season.

Figure 11 shows the average MAPE values of the five experiments for each season. Looking at the different seasons, the MAPE is higher in summer than in the other seasons. The main reason is that the load is higher and changes more dramatically in summer. After considering seasonal and temperature predictors, *S2* and *S4* showed better prediction accuracy than *S* and *S3*. The decrease of MPAE is very obvious, especially in spring and summer.

From the above analysis, the best prediction accuracy is obtained for the comparison experiment *S5* considering six periodic factors and three nonperiodic factors. Although a predictor improves the prediction accuracy only in some time periods, such as weekday factor for weekends, it still shows that the periodic factors and nonperiodic factors are important for electricity load forecasting.

## 5. Conclusion

In this paper, we analyze the data of electricity load in Tai'an, Shandong Province, from January 2016 to August 2018 to obtain the predictors. These include daily variation and weekly variation of electricity load, holidays and weekdays, and seasonal factors. A prediction model with six periodic factors and three nonperiodic factors was developed based on these four types of predictors. The experimental results show that the selected predictors all improve the accuracy of electricity load forecasting. It is considered that one predictor can significantly improve the prediction accuracy for certain dates. Meanwhile, the prediction results of experiment *S5* considering all predictors are better than those of the experiment considering some predictors. Experiment *S5* establishes an electricity load forecasting model based on multiple periodic predictors and nonperiodic predictors, which is applicable to the electricity load forecasting in Tai'an city.

In addition, it can be observed that the electricity load forecasting is more difficult for some specific moments. The more difficult moments of the day are 12 : 00, 13 : 00, and 18 : 00. The difficult part of the week is the weekend. A common point of these moments is the rapid variation in electricity load. The prediction accuracy of these moments is improved when we consider the predictors that cause load changes. For example, after adding the weekday factor, the prediction accuracy of the weekend was effectively improved.

Although the prediction accuracy in the specific moments is improved by adding the characteristic factors, there is still a difference compared with that in the stable moments. How to further improve the prediction accuracy at the moments of the load rapid variation will be the next research direction.

## Figures and Tables

**Table 1:** Specific dates of national statutory festivals in China.

Holidays	2016	2017	2018
New Year's Day	1.1–1.3	2016.12.31–2017.1.2	2017.12.30–2018.1.1
Chinese Lunar Spring Festival	2.7–2.13	1.27–2.2	2.15–2.21
Qingming Festival	4.2–4.4	4.2–4.4	4.5–4.7
May Day	4.30–5.2	4.29–5.1	4.29–5.1
Dragon Boat Festival	6.9–6.11	5.28–5.30	6.16–6.18
Min-Autumn Festival	9.15–9.17	10.1–10.8	9.22–9.24
Chinese Nation Day	10.1–10.7		10.1–10.7

**Table 2:** Results of five comparison experiments.

Experiment	*S*	*S1*	*S2*	*S3*	*S4*
MAE	35.46	34.66	35.13	34.76	33.88
MPE	0.62%	0.62%	0.64%	0.50%	0.69%
MAPE	4.29%	4.22%	4.26%	4.19%	4.11%

## Data Availability

The data used to support the findings of this study are included within the article.
